# Knowledge, Attitudes, and Practices regarding Avian Influenza (H5N1), Afghanistan 

**DOI:** 10.3201/eid1409.071382

**Published:** 2008-09

**Authors:** Toby Leslie, Julie Billaud, Jawad Mofleh, Lais Mustafa, Sam Yingst

**Affiliations:** Ministry of Public Health, Kabul, Afghanistan (T. Leslie, J. Mofleh, L. Mustafa); US Naval Medical Research Unit 3, Cairo, Egypt (T. Leslie, S. Yingst); London School of Hygiene and Tropical Medicine, London, UK (T. Leslie); Sayara Media Communication, Kabul (J. Billaud); United Nations Food and Agriculture Organization, Kabul (S. Yingst)

**Keywords:** H5N1, avian influenza, KAPs, knowledge, attitudes, and practices, socioeconomic status, Afghanistan, information education, communication, dispatch

## Abstract

From February through April 2007, avian influenza (H5N1) was confirmed in poultry in 4 of 34 Afghan provinces. A survey conducted in 2 affected and 3 unaffected provinces found that greater knowledge about reducing exposure was associated with higher socioeconomic status, residence in affected provinces, and not owning backyard poultry.

Avian influenza (H5N1) has been reported in southern Asia (*1*). In Afghanistan, avian cases were confirmed from February through April 2007 in 4 of 34 provinces (*1*). No human cases have been detected, although limited human-to-human transmission has been reported from Pakistan (*2*). Backyard poultry (chickens) were affected in 20 of 22 outbreak sites in 4 eastern provinces. No outbreaks have been reported from commercial facilities. The response in Afghanistan was to cull all poultry within a 3-km radius, restrict poultry movement and importation, and conduct intensive influenza-like illness surveillance and information, education, and communication (IEC) campaigns within affected provinces. IEC campaigns included leaflets distributed in affected areas and broadcast media coverage on local television and radio. The campaign was designed to inform the public through messages aimed at reducing exposure to disease, preventing spread in poultry, and encouraging reporting. Additional IEC messages were aired nationally and outbreaks were widely reported by local news media. We conducted a survey of knowledge, attitudes, and practices (KAPs) regarding avian influenza in Afghanistan. The aim was to assess factors associated with KAPs.

## The Study

Five provinces in Afghanistan were selected as a convenience sample (accessibility) that included both affected and unaffected areas. Two accessible districts in each province were randomly selected by using a random number generator. Random transects were used to select 10 households per village. To give an approximately equal male:female ratio, either the head of household, spouse (woman), or the oldest person available at the time was selected. Participants provided informed consent. Ethical approval was provided by the Institutional Review Board, Ministry of Public Health, Afghanistan.

A standardized, structured questionnaire collected information on demographic and socioeconomic measures, avian influenza information sources and knowledge of appropriate preventive measures, poultry and animal handling, food and generic hygiene, and human influenza knowledge and treatment seeking. Questions related to KAPs were scored by a panel of experts in related disciplines. The questions were ranked for importance in preventing avian influenza transmission in poultry or reducing human exposure and awarded 5 points, 3 points, or 1 point for correct answers. For each respondent, the sum of scores for correct answers divided by the sum of available points generated a percentage score. Blank responses to questions were counted as such and not included in individual denominators. The questionnaire was back-translated and pilot-tested. The survey was conducted in May 2007, by trained Afghan surveyors. Data were double-entered by using Microsoft Access (Microsoft, Redmond, WA, USA) and analyzed by using Stata 8 software (Stata Corporation, College Station, TX, USA).

KAP scores provided a weighted measure of KAPs related to prevention of avian influenza. Percentage scores for each respondent were ranked and classified as above or below the median. The primary analysis was conducted to compare factors (age, sex, socioeconomic status, provincial exposure to avian influenza IEC campaigns, and poultry ownership) associated with knowledge above the median. Socioeconomic quintiles (SEQs) were defined by principle components analysis using employment, education, and household assets as indicators (*3*). Factors independently associated by univariate regression at the 95% confidence level were included in a stepwise multivariate logistic regression model. To numerically evaluate KAP levels, a secondary analysis assessed differences between mean percentage scores, stratified by factors identified by logistic regression analysis.

Data for 304 respondents were included in the analysis. Of the 5 provinces, Kabul and Nangahar had had influenza outbreaks in poultry in 2007. Enrollment characteristics are shown in the Table. Median age of respondents (38 years) was high, but it reflected the age of heads of households and spouses. Poultry ownership was reported by 65.2% of households (>95% backyard ownership) and differed significantly between SEQs (poorest 53/62 [85.5%] vs. least poor 20/55 [36.4%]; χ^2^ 30.0, p<0.001).

**Table:** Enrollment data for avian influenza knowledge, attitudes, and practices survey, Afghanistan, May 2007

Characteristic	Value
No. respondents	304
% Male	46.8
Median age, y (interquartile range)	38 (27–50)
Age range, y, no. (%)*	
15–20	30 (10.0)
21–30	85 (28.2)
31–40	64 (21.3)
>40	122 (40.5)
No. (%) in each province	
Herat†	32 (10.5)
Kabul‡	64 (21.0)
Kandahar	79 (26.0)
Nangahar‡	64 (21.0)
Samangan	65 (21.0)
No. (%) with no formal education	
Male	36 (26.1)
Female	117 (75.0)

*Age data missing for 3 respondents. †Only 1 district reported results because of security concerns. ‡Provinces exposed to avian influenza and intensive information, education, and communication campaigns (Kabul, March 2007, and Nangahar, February 2007).

SEQ was positively associated with KAP score above the median (lowest vs. highest: adjusted odds ratio [AOR] 14.3, 95% confidence interval [CI] 5.2–39.9), as was provincial exposure to avian influenza IEC campaigns (AOR 9.5, 95% CI 4.9–18.6). Backyard poultry ownership (nonowners vs. owners: AOR 0.3, 95% CI 0.2–0.7) and older age group (15–20 years vs. >40 years: AOR 0.3, 95% CI 0.1–0.8) were both negatively associated.

For secondary analysis, overall mean KAP score was 44.4%. Mean KAP score differed between SEQ (p<0.001, by analysis of variance) and was higher in provinces previously exposed to IEC campaigns (50.2% vs. 40.1%; p<0.001, by *t* test).

Specific, self-reported practices also differed by SEQ. Reporting of sick or dead poultry to authorities was less frequent among lowest SEQ (8/47 [13%]) than highest SEQ (20/49 [37%]; χ^2^ 6.6, p = 0.02) where selling poultry in the event of a local outbreak was more commonly reported (21/66 [66%] vs. 10/51 [18%]; χ^2^ 27.2, p<0.001). Presence of coops was less frequent in lowest SEQ (9/49 [18.4%]) than in highest SEQ (21/46 [45.6%]; χ^2^ 8.2, p = 0.004).

## Conclusions

Human cases of avian influenza (H5N1) have resulted from contact between humans and infected backyard poultry (*4*). Risk to humans is also related to frequency of disease occurrence in the avian population (*5*). Recently, human-to-human transmission has been reported in the neighboring Northwest Frontier Province of Pakistan (*2*). Knowledge of disease is therefore a key factor in reducing exposure and enhancing reporting.

Overall knowledge was low, although in provinces exposed to intensive IEC campaigns, KAP scores of the population were higher. This finding indicated that campaigns had some success in increasing awareness. The level of concern generated by the campaign, government response, media reports, and proximity to the outbreak are all likely to contribute to this association. Despite this encouraging evidence, level of knowledge was far higher among persons with higher socioeconomic status. This finding contrasts with frequency of poultry ownership. Exposure risk is therefore likely to be considerably higher among lower socioeconomic groups.

Our results can be broadly generalized to the population, although we did not have access to unsafe districts (most of the districts in southern and eastern Afghanistan). This limitation may introduce selection bias, which would underestimate the effect of socioeconomic status because those living in inaccessible areas likely have a lower status than persons in accessible areas. Preintervention and postintervention surveys would provide a more robust measure of effectiveness. In the immediacy of an outbreak, this was unfeasible and would have been unethical. Although there are limitations to the study design in concluding intervention effectiveness, the results provide evidence to support further intensive campaigns as a response to influenza outbreaks in poultry.

Several reports have examined KAPs and behavior related to avian influenza (H5N1) (*6*–*9*). Similar to the finding in the Lao People’s Democratic Republic (*6*), our study suggests that conventional education and behavior change messages have a limited effect in populations with highest exposure. Efforts to ensure that IEC messages are suitable for lower socioeconomic groups should be adopted, specifically by improving the knowledge of community leaders, designing messages in a suitable format for the poor and illiterate, and ensuring that the most accessible channels are used. Messages should carefully balance the risk for human disease against potential nutritional and economic consequences of high population concern (e.g., food scares).

Successfully promoting behavior change is a lengthy process and requires frequent reinforcement. The acuteness of avian influenza (H5N1) outbreaks requires a concerted effort to enhance knowledge and change behavior among those most at risk in low-income countries.
